# Acetabular superior wall fractures: a distinct acetabular fracture entity

**DOI:** 10.1007/s00402-026-06407-z

**Published:** 2026-07-09

**Authors:** Axel Gänsslen, Dietmar Krappinger, Emmanouil Liodakis, Jan-Dierk Clausen, Tarek Omar-Pacha, Richard A. Lindtner, Stephan Sehmisch, David B. Osche

**Affiliations:** 1 Department of Trauma Surgery, Hannover Medical School, Carl Neuberg Str. 1, Hannover, Germany; 2 Department of Orthopaedics and Traumatology, Innsbruck Medical University, Innsbruck, Austria; 3 Department of Trauma, Hand and Reconstructive Surgery, Universitätsklinikum des Saarlandes, Homburg, Germany

**Keywords:** Acetabular fracture, Superior wall, Acetabular dome, Comminution, Hip instability, Clinical outcome

## Abstract

**Introduction:**

A transitional “grey-zone” exists between typical locations of classical trapezoidal anterior wall and large-fragment posterior wall acetabular fractures. Part of this grey zone is the superior acetabular dome, which is commonly involved in acetabular fracture patterns, especially in geriatric patients. The aim of this study was to characterize isolated acetabular superior wall fractures and to assess their relevance as a distinct fracture pattern.

**Methods:**

A retrospective analysis of pelvic fracture databases from three tertiary trauma centers was performed. Acetabular fractures with superior dome involvement were screened, and cases with isolated superior articular involvement were included. Overall, seven patients were identified. Clinical and radiological data were analyzed regarding fracture morphology, associated fracture characteristics (including dislocation, comminution, marginal impaction, intra-articular fragments, and femoral head injury), concomitant pelvic ring injuries, treatment, and clinical outcomes.

**Results:**

Isolated superior wall acetabular fractures occurred following both high-energy and low-energy mechanisms, including simple falls. The mean age was 48 years and four of seven patients were male. Hip dislocation or subluxation was observed in four cases (anterior-superior or purely superior). Surgical treatment was performed in six cases, predominantly using anterior approaches (mainly the iliofemoral approach) with screw and/or plate fixation. Outcomes were frequently unfavorable, with relevant joint degeneration or persistent symptoms, likely related to substantial articular damage of the superior weight-bearing zone.

**Conclusion:**

Acetabular superior wall fractures present a distinct fracture pattern that differs from classical anterior and posterior wall fractures and is less commonly observed. These injuries involve the superior weight-bearing dome and are frequently associated with comminution and substantial articular damage of both the acetabulum and femoral head. Even in cases with restored joint congruence, outcomes appear inferior, underscoring the clinical relevance of this fracture entity.

## Introduction

In the early 1960s, Judet and Letournel developed the still valid and widely accepted classification of acetabular fractures, based on a fundamental anatomical and radiological analysis of acetabular morphology according to the two-column principle [13, 14, 19, 20]. As early as 1981, they acknowledged that certain fracture patterns could not be adequately classified within their system of elementary and associated acetabular fracture types [19].

Already in 1981, Letournel distinguished between transitional and extra-articular fracture patterns beyond the classical ten fracture types [19, 20]. Transitional fractures represent a „grey-zone“ between established fracture entities and may be considered intermediate forms linking two distinct fracture patterns.

For instance, injuries to the quadrilateral plate (QLP) were not included in the original Letournel classification. More recently, a “three-column” classification has been proposed, particularly addressing acetabular fractures in elderly patients, incorporating an anterior column, a posterior column, and an additional roof column [34].

Anterior wall fractures (AW) are rare [20]. Detailed descriptions include anterior-superior fracture variants, which can be associated with an anterior hip dislocation and frequently present as avulsion fractures of the anterior inferior iliac spine (AIIS) [6, 22, 30]. Lenarz et al. further differentiated various anterior wall fracture entities [18], including classical AW fractures (trapezoidal fragment), anterior-superior rim fractures, superior iliac wing fractures with articular involvement, classified by Letournel as posterior wall (PW) fractures [20] and intra-articular AIIS avulsion fractures.

Notably, superior fracture patterns correspond to injuries of the acetabular dome area, which have been described as “third column” fractures by Zhang et al. [34]. This proposed third column acts as a keystone between the anterior and posterior columns in the Letournel classification. The corresponding acetabular roof region has been termed the “roof wall“ [34]. Restoration of all three walls—anterior, posterior, and superior wall (roof wall) - is considered to be of major clinical importance [34]. In addition, a medial wall exists, largely formed by the QLP [7].

Letournel described two cases of complete detachment of the roof as part of a large fragment of the anterior iliac wing. As no disruption of the pelvic brim (iliopectineal line) was present, these injuries were classified as PW fractures. He interpreted this fracture pattern as a transitional form between anterior column fractures and PW fractures [20].

More recently, Giannoudis and co-workers subdivided AW fractures into inferior, intermediate, and superior subtypes, based on a topographical division of the anterior wall into three segments. Superior AW fractures were defined as those involving the proximal third of the anterior wall [29]. Based on this definition, these fractures are located adjacent to the anterior inferior iliac spine (AIIS) and involve the acetabular dome.

Involvement of the acetabular dome is a common feature in acetabular fracture pathology, particularly in geriatric patients. Superior marginal impaction typically results from a simple fall from standing height with the hip joint in extension and neutral rotation [11, 15, 28]. In this setting, force transmission occurs via the greater trochanter to the femoral neck and head [15], while femoral neck anteversion directs the primary force vector toward the anterior and superior acetabulum [32]. In contrast, purely superiorly directed mechanisms leading to acetabular fractures are rare.

Although roof wall fractures are categorized within the three-column classification system (type A3.1 injury), isolated superior wall fractures are not defined as a distinct clinical entity, and their characteristics and clinical implications remain poorly understood. The aim of the present study was to analyse a series of isolated superior wall fractures of the acetabulum, characterize their morphology and associated injuries, describe treatment strategies, and assess their clinical outcomes.

## Materials and methods

Based on case discussions on specific acetabular fracture types during dedicated pelvic trauma courses, a retrospective database analysis was performed across three tertiary trauma centers. Institutional pelvic fracture databases from the Department of Trauma Surgery at Hannover Medical School (Germany), University Hospital Innsbruck (Austria), and Saarland University Hospital Homburg (Germany) were screened for anterior column (AC), AW, and PW fractures. All fractures with involvement of the superior acetabular dome were reviewed, and cases with isolated superior articular involvement were selected for further analysis.

A total of seven patients met the inclusion criteria. These patients were analyzed with regard to demographic characteristics, mechanism of injury, and clinical course. Radiological imaging data were reviewed using the institutional picture archiving and communication systems (PACS) to assess fracture classification, associated fracture characteristics (including type of dislocation, comminution, marginal impaction, intra-articular fragment, and femoral head injuries), concomitant pelvic ring injuries, and definitive treatment.

This retrospective study was conducted at these university hospitals between January 2008 and May 2025. Ethical approval was obtained from the local ethics committee (Nr. 12361-BO-K-2026).

## Case analysis

Seven patients with isolated superior wall fractures involving the acetabular dome area were identified.

### Case 1

A 37 year-old male patient sustained a 4 m fall with axial forces acting on the left acetabulum. Severe supraacetabular displacement was detected on CT-evaluation without signs of hip dislocation. No associated injuries were present. On day 7, ORIF was performed with screws and a curved superior dome reconstruction plate via Smith-Petersen approach. Intraoperatively, no femoral head (FH) lesion was seen, but cartilage contusion and impaction at the superior acetabular dome was present with comminution of the superior acetabular rim. Within 5 months, superior FH subluxation developed, leading to secondary THR after 6 months.

### Case 2

A 66 year-old female patient sustained a 3 m fall with axial forces acting on the left acetabulum. Severe supraacetabular displacement was detected on x-rays and CT-evaluation with signs of superior hip subluxation. No associated injuries were present. On day 6, ORIF was performed via the Smith-Petersen approach with screws and a vertically orientated reconstruction plate. Intraoperatively, femoral head cartilage contusion and acetabular articular impaction was observed at the superior acetabular dome area. At latest follow-up, 62 months after trauma, she complaint of low back pain as prior to injury but without inguinal or fracture-related pain. Radiographically, grade III degeneration was seen with relevant joint space narrowing and osteophytes.

### Case 3

**Fig. 1:**
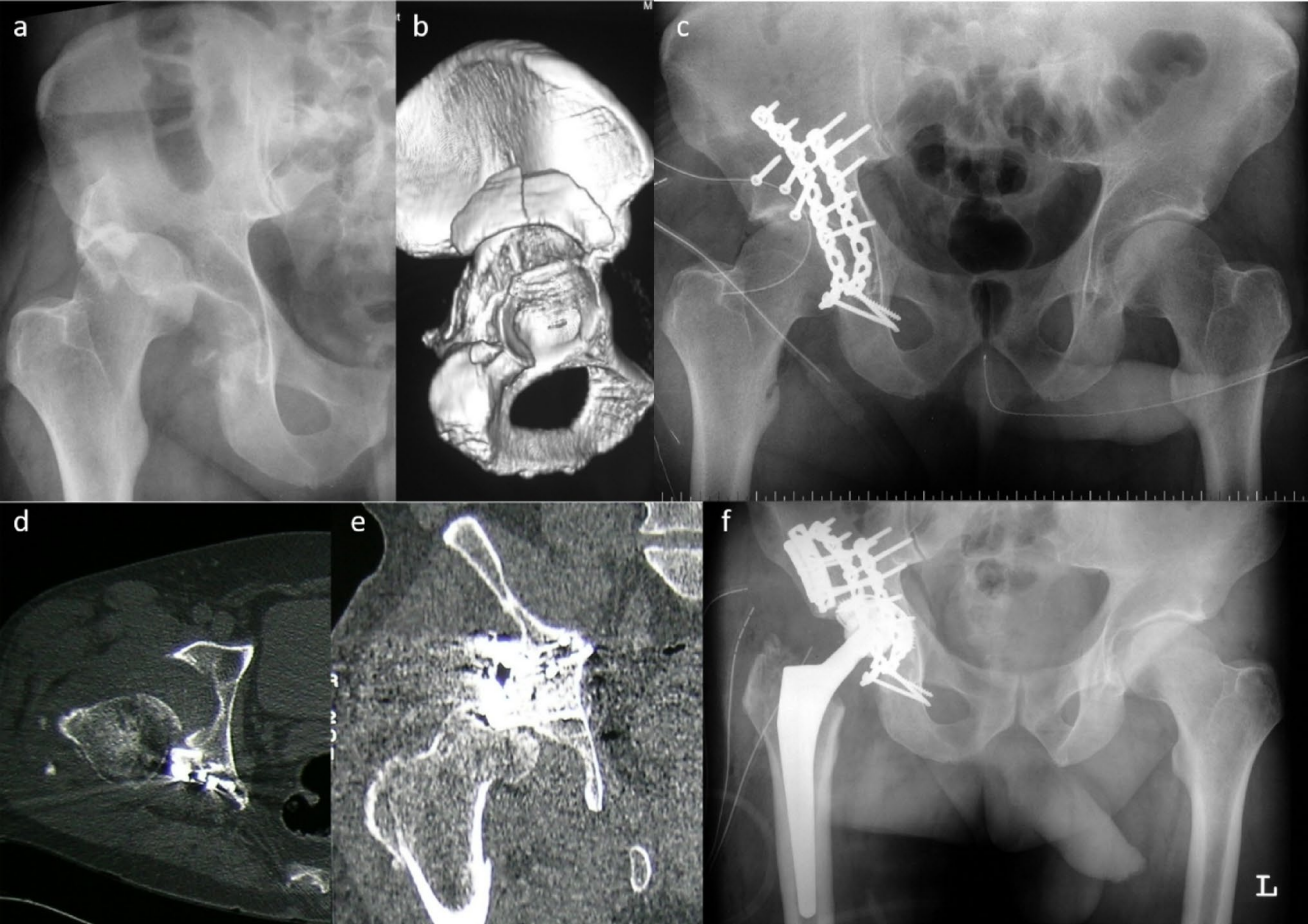
Case of a complete superior wall fracture consisting of two large fragments (for details: see text).

A 42 year-old male patient sustained a motorcycle accident while colliding with a tree (dashboard mechanism) (Fig. 1). The injury mechanism was proposed of having the hip in external rotation and approximately in 50° flexion. Additionally, an ipsilateral tibial head fracture was present. The femoral head dislocated cranially (a), and closed reduction failed. CT-evaluation revealed a large superior wall fracture consisting of two main fragments in association with a small posterior wall fragment and supraacetabular comminution with a superior acetabular defect (b).

ORIF was performed using the Kocher-Langenbeck approach on day 1 with screw fixation of the superior fragments and double posterior/posterior-superior plate fixation (c). Intraoperatively, marginal impaction of the FH and FH cartilage contusion were detected. Corresponding to these lesions, acetabular cartilage contusion, impaction and local comminution were present.

Within 6 weeks, superior FH re-dislocation developed with AVN of the FH (d, e), resulting in early THR (f).

### Case 4

**Fig. 2:**
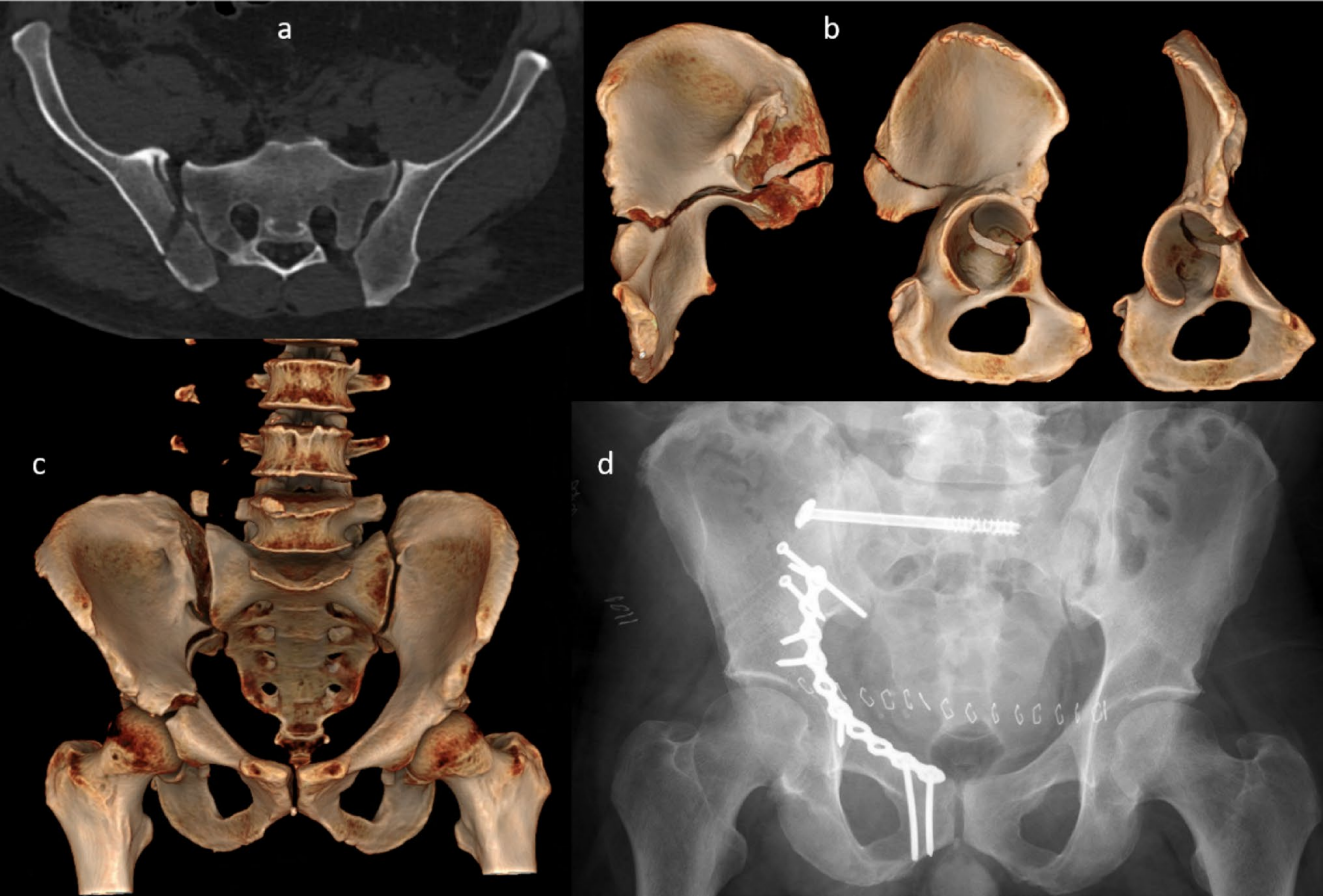
Case of an unusual supraacetabular horizontal fracture associated with a transiliac fracture dislocation with complete superior dome involvement (for details: see text)

A 51 year-old male patient sustained multiple injuries after a fall from 4 m height onto his right body (Fig. 2). He sustained a moderate chest trauma with multiple rib fractures and a hematopneumothorax, several transverse and spinal process fractures of the thoracic and lumbar spine, a blunt abdominal trauma with a subcapsular liver contusion and a small superficial laceration and a right acetabular fracture with SI-joint injury (ISS = 34 points). He was hemodynamically stable and was transferred on day 4 to our hospital. An unusual supraacetabular horizontal fracture with involvement of the SI-joint (transiliac fracture dislocation) with complete superior dome involvement was seen (a, b,c). On day 11, definitive fixation of his injury was performed. An extended intrapelvic approach was used (additional 1st window) and suprapectineal plating was performed after adequate reduction. The disrupted SI-joint was stabilized with a percutaneously inserted iliosacral screw (d). Complete joint congruence was achieved and anatomic pelvic ring reconstruction.

At latest follow-up (9 m), hip rotation and hip flexion was reduced and severe subjective pain was reported at the right distal flank area (Merle d`Aubigné score: 10 points). Radiographically, anatomic healing was seen.

### Case 5

68 year-old female after simple ground-level fall in her garden. She walked to the nearest hospital and was then transferred to a Level 1 trauma center after radiographic diagnostic. A multifragmentary superior wall fractures was diagnosed with anterior and superior hip subluxation. After 2 days surgery was performed using a Smith-Petersen approach. Intraoperatively, a small articular fragment was removed due to insufficient fixation. Acetabular superior marginal impaction was reduced against the reduced FH and supported by cancellous bone. Definitive fixation was done using screws. In maximal external rotation a slight subluxation occurred requiring capsular anchor re-fixation, resulting in a stable joint.

At 7 month follow-up, she experienced residual groin discomfort during activities involving external rotation. A centered FH was seen on the pelvic a.p. x-ray. A slight FH impaction was observed in the weight bearing superior area.

### Case 6

**Fig. 3:**
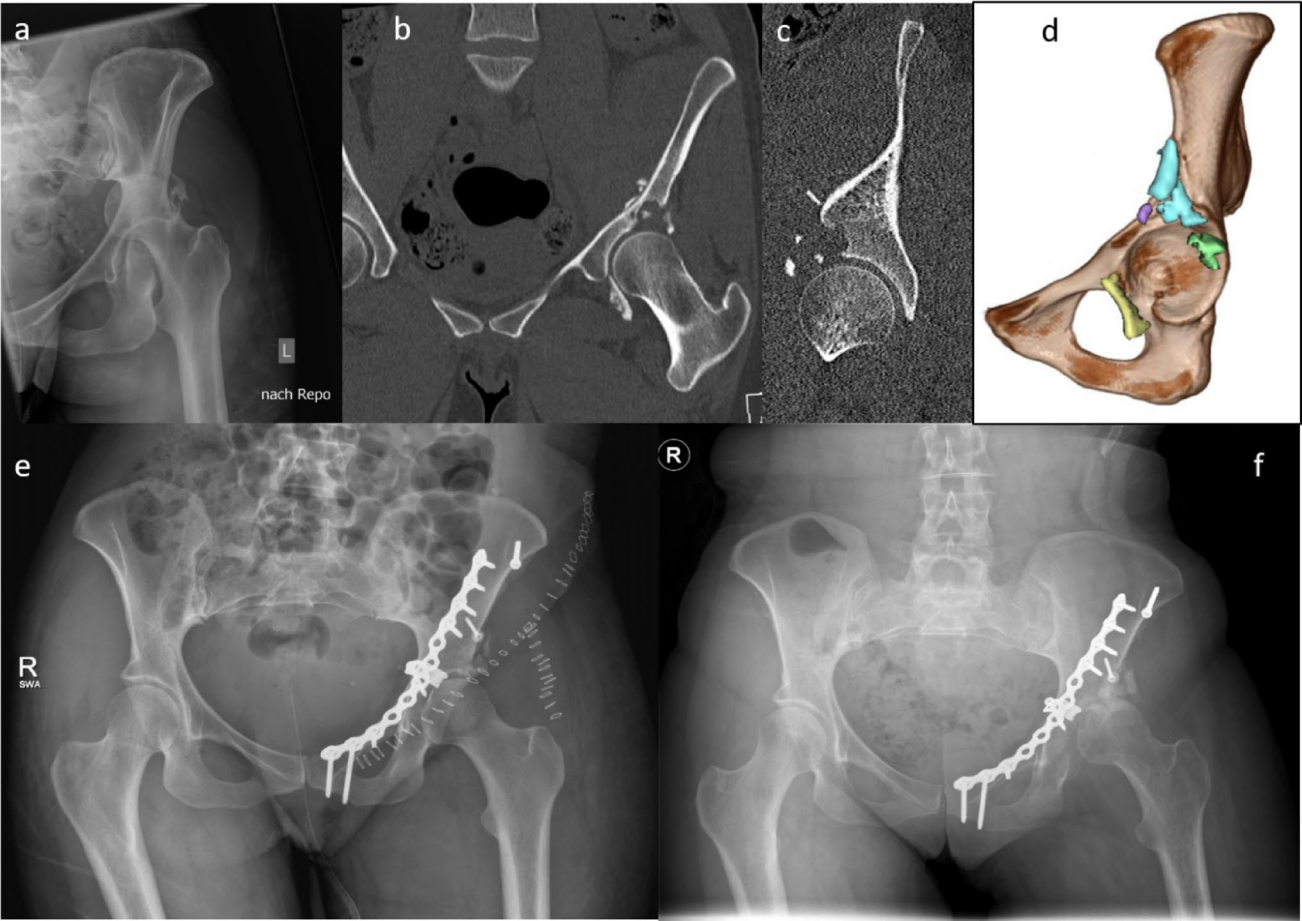
Case of an anterior-superior hip dislocation with a comminuted superior wall involvement (for details: see text).

A 34-year-old female sustained a low-energy fall while walking (Fig. 3). On admission, an anterior-superior hip dislocation was diagnosed (Fig. 3a). Two attempts at closed reduction failed before successful reduction was achieved. CT demonstrated multiple small fragments with a large superior acetabular defect and an associated anterior wall rim fragment (Fig. 3b, c, d).

Surgical treatment was performed on day 4 using a combined ilioinguinal and iliofemoral approach with anterior superior iliac spine osteotomy. Fixation included suprapectineal plating, a spring plate for the anterior rim fragment, and an attempted screw fixation of a larger supra-acetabular fragment (Fig. 3e). Postoperative radiographs and CT demonstrated a reduced and congruent hip joint.

At 6 months, superior subluxation of the femoral head and suspected necrosis of the anterior wall fragment were observed (Fig. 3f), and the patient was scheduled for THR.

### Case 7

**Fig. 4:**
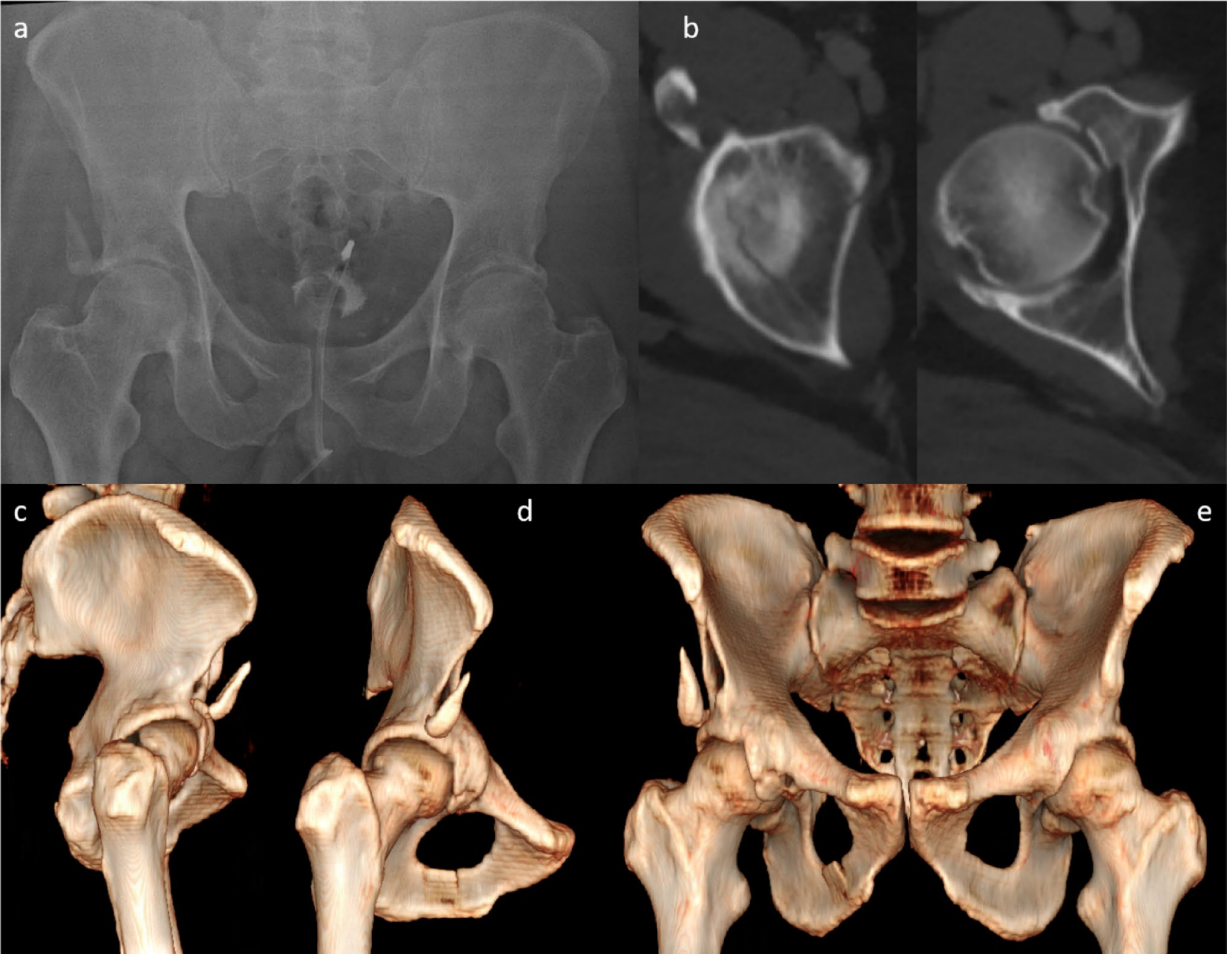
Case a displaced AIIS avulsion fracture with undisplaced fracture lines extending into the superior acetabular dome (for details: see text)

A 40-year-old male sustained polytrauma after being struck by a car at approximately 50 km/h as a pedestrian (ISS 41). Associated injuries included moderate traumatic brain injury, midface fractures, thoracic trauma with multiple left rib fractures and hemopneumothorax, a spinal epidural hematoma at the C1/2 level, B2-type injuries at C7/TH1 and Th4/5, and anterior cruciate ligament rupture. CT demonstrated an AIIS avulsion fracture (Fig. 4a) with fracture lines extending into the superior acetabular dome (Fig. 4b).

Non-operative treatment was pursued, as no intraarticular pathology was identified on CT (Fig. 4c–e).

At 4-year follow-up, the patient remained asymptomatic with no clinical complaints.

## Discussion

Physiological and biomechanical data highlight the relevance of the superior acetabulum (i.e., the acetabular dome). The hip joint exhibits a physiological incongruence due to a mismatch between the diameter of the femoral head and that of the acetabulum, with the femoral head being slightly larger [1]. Consequently, initial joint loading occurs in the anterior and posterior regions of the lunate surface, followed by loading of the acetabular dome [8, 9]. The highest forces acting on the acetabulum are concentrated in its superior region, the acetabular dome [16, 31], corresponding to the area of greatest acetabular cartilage thickness [17]. Finite element analyses support these findings, demonstrating peak stresses in the superior acetabular region during activities such as standing up and walking [23, 33]. Age-related differences in subchondral mineralization of the lunate surface have been described. In younger individuals, peak loading is distributed between the anterior and posterior regions of the lunate surface, whereas in elderly patients, maximal subchondral density is concentrated at the apex of the lunate surface [25]. This shift may be attributed to increased stiffness of the acetabulum associated with degenerative changes.

Only few reports of superior acetabular fractures are available in the literature. Letournel described a fracture pattern with a superior wall fragment occurring as part of a both-column fracture [14]. In the latest textbook, he characterized the superior injury pattern as a “pure superior fracture completely above the boundary of the posterior border and posterior wall but involving the upper part of the anterior lip of the acetabulum” [20]. As the iliopectineal line and the obturator ring showed no fracture extension on radiographs, this fracture type was subsumed under posterior wall fractures. He observed two such fractures (2 of 940 cases; 0.2%) [20].

Richards et al. presented a case of an isolated superior acetabular fracture and described it as a rare posterior wall variant [27]. In their definition, this fracture consists of a superior roof fracture associated with an anterior iliac wing fracture. A minimally displaced case with preserved congruency of the superior acetabular roof was reported. CT images demonstrated a typical anterior column fracture line terminating at the anterior-inferior acetabular horn. ORIF was performed using supra-acetabular screw fixation, and the iliac wing fracture was stabilized with a reconstruction plate at the iliac crest. The ilioinguinal approach was used, with exposure limited to the first and cranial part of the second window. Intraoperatively, a 2-mm cartilage lesion of the superior aspect of the femoral head was identified. At 2-year follow-up, the Merle d’Aubigné score was 14 points, indicating moderate pain, while radiographs demonstrated anatomical healing.

In 2022, Desauge et al. analyzed three-column acetabular fractures, defined as sagittal comminution fractures involving a large portion of the acetabular dome with a characteristic triangular morphology [5]. Using the ilioinguinal or intrapelvic approach, fixation of the independent roof fragment was not achievable in their series.

Paulraj et al. focused on posterior-superior wall fractures and identified 20 cases, with a 70% rate of posterior hip subluxation or dislocation [24]. Fifteen patients were treated surgically by open reduction and screw plus plate fixation. No intermediate- or long-term outcomes were reported.

Recently, Haider et al. reported a case of a displaced acetabular roof fracture associated with a multifragmentary iliac wing fracture without pelvic ring disruption [10]. Treatment consisted of delayed screw fixation (> 2.5 weeks post-trauma), performed via an iliofemoral approach with ASIS osteotomy. Postoperative CT demonstrated a residual articular gap. At 6-month follow-up, adequate hip function was reported (Merle d’Aubigné score: 17).

The cases presented in this manuscript differ with respect to both injury mechanism and fracture morphology. While superior wall fracture patterns have predominantly been described in the context of high-energy trauma, our series demonstrates that similar fracture configurations may also occur following low-energy mechanisms, such as simple falls. Notably, even in these cases, significant joint instability, including hip subluxation or dislocation, was observed. As expected, axial loading mechanisms following falls from greater heights (3–4 m) were associated with these fracture types. However, only two patients sustained polytrauma, one patient had a single associated injury, and four patients presented with isolated acetabular fractures.

We observed highly heterogeneous fracture patterns, which can be categorized as follows:

- Type 1: displaced AIIS avulsion fracture with fracture lines extending into the superior acetabular region (without displacement of the articular component in our case).
- Type 2: posterior pelvic ring injury (transiliac fracture-dislocation) with superior articular involvement.
- Type 3: multiple large superior wall fragments with complete detachment of the superior articular surface.
- Type 4: comminuted superior acetabular margin fracture consisting of multiple small fragments.

Type 1 injuries resemble AIIS avulsion fracture patterns recently described by Meena et al. [21]. In their series, six patients sustained high-energy resulting in AIIS avulsion fractures, in some cases extending to the ASIS, and associated hip instability. These injuries were treated surgically using screw fixation of the anterior ilium via an iliofemoral approach. One patient presented with an AIIS avulsion fracture in association with a transverse acetabular fracture. In contrast, extraarticular AIIS avulsion fractures are predominantly managed non-operatively, even in cases with displacement. However, malunion may result in extra-articular femoroacetabular impingement [26], and careful 3D evaluation is recommended, with a displacement threshold of approximately 1.5–2 cm suggested in the literature [3]. In contrast to our case, all patients in the series by Meena presented with associated superior (anterior) hip subluxation or dislocation. In one case, a large intraarticular fragment was present. Only three cases were described in detail, two of which presented with anterior hip dislocation. The radiograph of the third case corresponds more closely to our type 2 injury pattern, although without extension to the posterior pelvic ring. Based on the presented radiographs, no uniform fracture pattern could be identified. All six patients had good to excellent long-term outcomes after a minimum follow-up of 42 months.

In the type 2 case (case 4), a different injury mechanism is assumed. A lateral compression mechanism with direct force transmission to the iliac wing resulted in an iliac fracture dislocation with superior articular involvement. This injury type corresponds to an extreme iliac wing fracture. Extra-articular comminuted iliac wing fractures typically occur following high-energy trauma [12], and open injuries are common [4]. Richards et al. described a similar fracture pattern without sacroiliac joint involvement after direct high-energy trauma to the lateral pelvis [27]. In their case, the fracture remained confined to the superior region, sparing the iliopectineal line, ilioischial line as well as the obturator foramen, and ascending into the iliac crest, where it remained incomplete. As described above, intraoperative findings included a small full-thickness cartilage lesion of the cranial femoral head, and fixation was performed using a combination of screw and plate techniques. The reported outcome was fairly good (Merle d’Aubigné score: 14; Harris Hip Score: 73). In this fracture type, the degree of joint destruction appears less pronounced than in other types, as observed in our cases. A comparable fracture morphology has also been illustrated in a bone model by Lenarz et al.; however, no corresponding clinical case was reported [18].

The fracture pattern in our case 3 most closely corresponds to an atypical posterior wall fracture, as described by Letournel [20]. Complete disruption of the superior acetabulum with large fragments and full articular involvement suggests the application of high forces to this region. Given the biomechanical relevance of the superior wall area, joint instability and associated articular injuries—such as supra-acetabular comminution, marginal impaction of the femoral head, cartilage contusion, and acetabular articular impaction—likely contributed to early joint failure.

Lenarz et al. reported six atypical AW fractures, involving the anterior acetabular rim but without involvement of the pelvic brim. All fractures were associated with anterior hip dislocation and demonstrated hip instability and/or joint incongruity [18]. Their schematic fracture model showed fracture extension into the AIIS and interspinous region, indicating combined intra- and extra-articular involvement, while remaining inferior to the level of the ASIS. Notably, involvement of the true superior weight-bearing dome—corresponding to the region of the gluteus medius pillar—was not described, indicating that these fractures predominantly affected the anterior-superior acetabular region rather than the superior dome itself. In their clinical series, all six patients (mean age 27 years) were treated surgically via a modified Smith-Peterson approach using screws and short plates. It was noted, that femoral head chondral lesions, intraarticular comminution and marginal impactions were observed in nearly all cases, frequently accompanied by labral tears. The five patients with a minimum follow-up of 1 year showed good and excellent clinical and radiographical results.

In addition, Saracco et al. performed a literature review on AW fractures and proposed a topographical classification system [29]. In this system, the AW is divided into superior (region below the AIIS), intermediate (iliopectineal eminence region), and inferior (region adjacent to the pubic ramus) segments. Notably, involvement of the “superior” region in this classification reflects anterior-superior wall involvement rather than the true superior weight-bearing dome. Nevertheless, fractures of the superior AW may reasonably be considered within the spectrum of superior wall fractures. Furthermore, a longitudinal subdivision based on the extent of wall involvement was introduced, distinguishing peripheral-lateral (< 25% wall involvement; type I), peripheral-central (25–50% wall involvement; type II), and peripheral-medial (> 50% wall involvement; type III) AW fractures. Only the latter may involve the iliopectineal line and thereby corresponds to classical anterior wall fractures according to Letournel, whereas type I and type II represent more peripheral fracture patterns. The reported cohort consisted predominantly of male patients (79.3%) with a median age of 37 years, most commonly following high-energy trauma (75%). Surgical treatment was performed using various anterior approaches with different fixation strategies [29].

In contrast to the findings by Lenarz et al., clinical outcomes in our cases 1, 2, 5, and 6 were considerably worse. One patient required early conversion to THR, while another was scheduled for THR 6 months after trauma. The two remaining patients demonstrated relevant joint degeneration or femoral head impaction. Possibly, this is due to a more superior joint injury (superior wall) with all patients showing severe comminution at the superior weight bearing area with resulting bony and articular defects, despite postoperative joint congruence. Additionally, our patients were older (mean age 48.8 years). Alos, the injury mechanism differed as a 34 old patient and a 68 year old patients suffered their injury by simple falls.

Lastly, it is important to take into account if hip subluxation or dislocation was present at the time of injury. Similar to posterior wall fracture-dislocations, these combined injuries place the femoral head at substantial risk and are associated with poorer clinical results. Similar to posterior wall fractures, intra-articular fragments may be entrapped after closed reduction, potentially leading to fragment necrosis despite temporary traction prior to definitive fixation. This was observed in one of our cases, in which repeated closed reduction was required and a large intra-articular fragment had to be retrieved surgically. At the time of admission of the patient to the reference hospital, a large articular fragment was trapped in the inferior part of the joint, which had to be recovered during surgery. Despite good containment of the femoral head and a centered hip joint with sufficient reduction of the superior part of the anterior wall on postoperative CT, necrosis of the wall fragment developed within 6 months. Comparable findings have been reported by Bastian et al., who described AW involvement in three of ten patients with anterior hip dislocation, with only fair outcomes (Harris Hip Score: 43 and 72 points) in two of these three cases at mid-term follow-up [2].

**Fig. 5:**
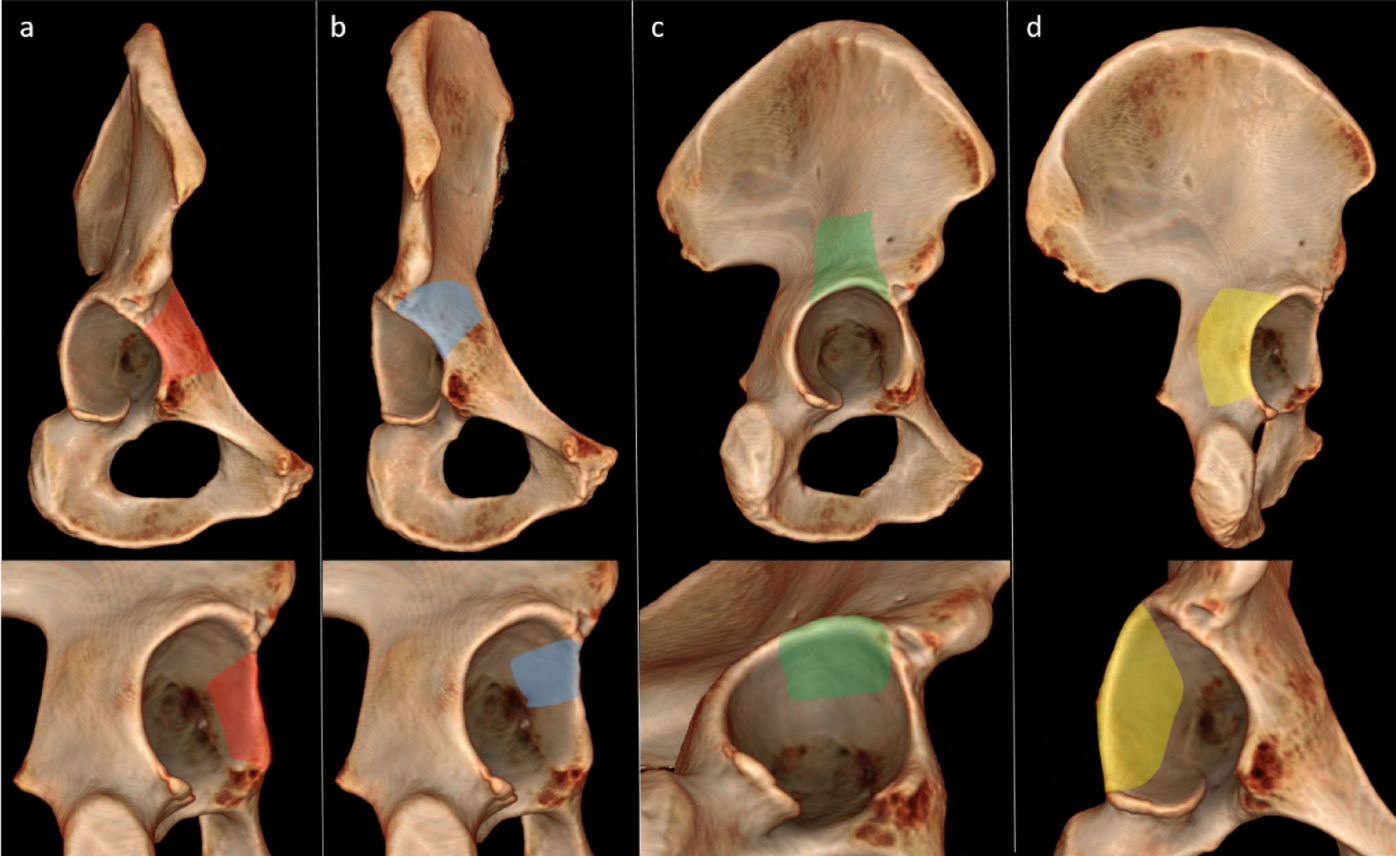
Concept of wall involvement of the acetabulum: pure anterior wall region **(a)**, anterior-superior wall region **(b)**, superior wall region **(c)**, posterior and posterior-superiorwall region **(d)**.

The key distinguishing feature of the fractures observed in our series is the involvement of the true superior wall and acetabular dome. Based on this observation, a morphological subdivision of the acetabulum into four distinct zones can be proposed (Fig. 5):

- *Pure anterior wall region* (Fig. 5a): fractures at the level of the iliopectineal eminence involving the anterior horn (approximately the 2–4 o´clock localization in the left hip); these correspond to inferior and intermediate fractures according to Saracco et al. [29].
- *Anterior-superior wall region* (Fig. 5b): fractures located in the area of the grove between the iliopectineal eminence and the AIIS; these fractures more closely correspond to fracture patterns described by Lenarz et al. [18].
- *Superior wall region* (Fig. 5c): these true superior wall fractures predominantly involve the superior weight bearing area (acetabular roof according to Letournel [20]); morphologically, the main fracture component is located posterior to the AIIS pillar, where the gluteus medius pillar reaches the superior acetabulum.
- *Posterior and posterior-superior wall region* (Fig. 5d): corresponding to classical posterior wall fractures as described by Letournel [20].

Following this morphological framework, superior wall fractures reflect higher force transmission through the weight-bearing dome, particularly involving the gluteus medius pillar, compared to other acetabular regions. Consequently, more severe articular damage is observed, including comminution with multiple small, non-reconstructable fragments resulting in bony defects, cartilage injury of both the femoral head and the acetabulum, and predominantly superior acetabular marginal impaction. This increased degree of articular destruction may explain the inferior clinical and particularly radiological results in our series.

## Conclusion

The present study demonstrates that acetabular superior wall fractures represent a specific and under-recognized fracture pattern, distinct from classical anterior and posterior wall fractures, although less commonly observed. These injuries are characterized by involvement of the weight-bearing acetabular dome, frequently presenting with displacement, comminution, and substantial articular damage affecting both the acetabulum and the femoral head. They may occur not only after high-energy axial loading mechanisms but also following low-energy trauma. Due to associated hip joint instability, including femoral head subluxation or dislocation, and fragment displacement, surgical management is the standard of care. Anterior approaches, such as the iliofemoral or Smith-Peterson approach with or without ASIS osteotomy, allow adequate exposure and fixation. Despite appropriate treatment, clinical and particularly radiological outcomes appear inferior compared to other acetabular fracture patterns, likely reflecting the critical involvement of the superior weight-bearing zone.

## Data Availability

No datasets were generated or analysed during the current study.

## Declarations

### Use of artificial intelligence

Artificial intelligence (AI) was used in the preparation of this manuscript solely to improve clarity and language. Specifically, the GPT-4 language model by OpenAI was used to assist in refining the text, enhancing readability, and ensuring grammatical accuracy. AI was not used for study design, data collection, data analysis, or interpretation.
